# Changes in age distribution of hemorrhagic fever with renal syndrome: an implication of China’s expanded program of immunization

**DOI:** 10.1186/1471-2458-13-394

**Published:** 2013-04-26

**Authors:** Xiaozhou He, Shiwen Wang, Xiaoxia Huang, Xiaofang Wang

**Affiliations:** 1 National Institute for Viral Disease Control and Prevention, China CDC, Beijing, China

**Keywords:** Hemorrhagic fever with renal syndrome, Expanded program of immunization, Age distribution, China

## Abstract

**Background:**

Vaccination against hemorrhagic fever with renal syndrome (HFRS) has been applied successfully for more than 20 years in China, and since 2008, the government has implemented the Expanded Program of Immunization (EPI) in regions with high incidence. In this study, we analyzed the EPI-related changes in age distribution in reported cases of HFRS and proposed new recommendations for prevention and control of the disease.

**Methods:**

Data relating to incidence of HFRS, geographical location and age distribution were collected through the China Information System for Disease Control and Prevention (CISDCP) from 2005 to 2010. Excel and SPSS 18.0 software, *χ*2 tests and descriptive methodology were used for analysis of the data.

**Results:**

A total 75 434 HFRS cases were reported in 28 provinces in China between 2005 and 2010. The majority of HFRS cases occurred in adults aged 30 to 55 and this group accounted for 68.3% of the total. With the implementation of the immunization program, HFRS age distribution has clearly changed in recent years. The proportion of HFRS cases among individuals targeted by EPI (16–60 years of age) decreased from 86.9% in 2005 to 81.9% in 2010. However, the proportion of cases among the non-vaccinated group aged <16 and >60 had increased from 13.1% in 2005 to 18.1% in 2010. Notably, in the >60 age group the proportion rose from 8.8% in 2005 to 14.7% in 2010. These differences were statistically significant.

**Conclusion:**

HFRS vaccination has played an important role in HFRS control and prevention in China. However, since the proportion of HFRS cases over 60 years old has increased significantly since EPI was implemented, it is recommended that the age limit for vaccination be reconsidered. This finding may have practical implications for more effective HFRS vaccination in the future.

## Background

Hemorrhagic fever with renal syndrome (HFRS), a rodent-borne viral disease caused by Hantaviruses, is characterized by fever, hemorrhagic manifestations and renal dysfunction [1,2]. The Hantaviruses are usually transmitted to humans via contact with rodent excretions such as saliva, feces and urine [1]. There are two main species of Hantavirus in China: the Hantaan virus (HTNV) and the Seoul virus (SEOV) [2]. China has reported over 1.5 million cases of HFRS since 1950 [3], and HFRS was classified as a Class B notifiable communicable disease in China in 1989. Since then, the number of annual cases throughout the country has been recorded in the national health database that is available for epidemiological study. Up until the end of 2010, all provinces of China, except Hainan province, had reported HFRS cases [4-6]. The annual number of cases in China during the last two decades constituted approximately 90% of the total cases reported worldwide. But the number of HFRS cases in China has experienced a steady decline in recent years [5,7]. By 2009, the nationwide number of reported cases (8842 cases) had reached its lowest level in nearly two decades.

Aimed at two species of Hantavirus (HTNV and SEOV), a safe and effective bivalent HFRS vaccine was developed in the 1990s [8] and has been successfully applied [9-12]. In 2008, the HFRS-targeted Expanded Program on Immunization (EPI) was implemented by the Chinese Central Government in order to reduce the HFRS incidence further. Free vaccine was provided to the seven provinces with the highest incidence, namely Heilongjiang, Jilin, Liaoning, Shandong, Hebei, Shaanxi and Zhejiang. One year later, in 2009, HFRS rates were found to have significantly decreased in EPI regions, and the vaccination-based program was further expanded to 10 other provinces: Hunan, Jiangxi, Inner Mongolia, Hubei, Henan, Jiangsu, Fujian, Anhui, Sichuan and Guangdong. As the number of cases among the 16 to 60 age group accounted for more than 80 percent of the total [13,14], and because the Pharmacopoeia of People’s Republic of China (2005) [15] specified that the vaccines could only be used in persons between 16 and 60 years old, the program focused on this age group [16]. However, the bivalent HFRS vaccine is also considered safe for other age groups [10].

In this study, we analyzed the reported HFRS case data from all 17 EPI regions and analyzed the changes in age distribution among the group targeted by EPI (16–60 years of age) and in the non-targeted groups (<16 and >60 years of age).

## Methods

### Data collection and statistical analysis

Data were obtained from the China Information System for Disease Control and Prevention (CISDCP) and provincial monitoring reports of previous years. This system, established in 2004, is a web-based reporting system of nationally notifiable infectious diseases and public health emergencies throughout the country [17,18]. The HFRS cases were diagnosed in the hospital or the local CDC according to the Diagnostic Criteria for Epidemic Hemorrhagic Fever. Two types of cases were analyzed in our study; clinically diagnosed patients and laboratory confirmed cases. Clinical diagnosis criteria included: exposure history (i.e. direct or indirect exposure to rodents and their excreta and saliva within two months prior to onset of illness); and acute onset of disease with at least two clinical symptoms (i.e. hemorrhagic fever, and one or more of the signs of hypotension, oliguresis or renal damage). Laboratory confirmed cases were diagnosed as positive using one of the relevant laboratory tests (HFRS IgM antibody positive, 4 fold rise of IgG antibody between acute and convalescent phage, Hantavirus RNA positive or isolation of Hantavirus from the patient) [19]. Individual case histories of the HFRS patients were collected via the CISDCP according to the Nationwide Surveillance Program for HFRS (try out) [20]. We obtained the following variables for HFRS cases from 2005 to 2010: gender, date of onset, date of birth and residential address.

Analysis was carried out in Microsoft Excel. We calculated the age at onset by using date of onset and date of birth. Then we categorized the data into <16 years, 16–60 years and >60 years age groups by province. HFRS annual incidence per 100 000 population for the nation and each province were collected from the Annual Epidemic Report which is provided by CISDCP as the Summary Analysis of the Epidemic. Demographic data was collected from the Basic Information System, a subsystem of the CISDCP. The number of cases was evaluated by the *χ*2 test in SPSS software to determine the statistical significance of the differences in proportions observed before and after the introduction of the vaccine. Since the coverage of CISDCP was not fully extended to the township level in some western parts of China, only 82.2% of township hospitals were covered in 2008 [21]; at the time of writing more than 85% have now been covered but these data are unpublished. Although some cases may not be reported, the evaluations on the quality of the reporting system have shown that the effect of missing reports has been reduced as much as possible [22,23].

### Database access permission statement

The initial disease data which were obtained from the China Information System for Disease Control and Prevention (CISDCP) that is open to the staff of China CDC. All the authors were authorized to obtain and analyze these data, and to publish the results.

## Results

### HFRS age distribution in china from 2005 to 2010

**Figure 1:**
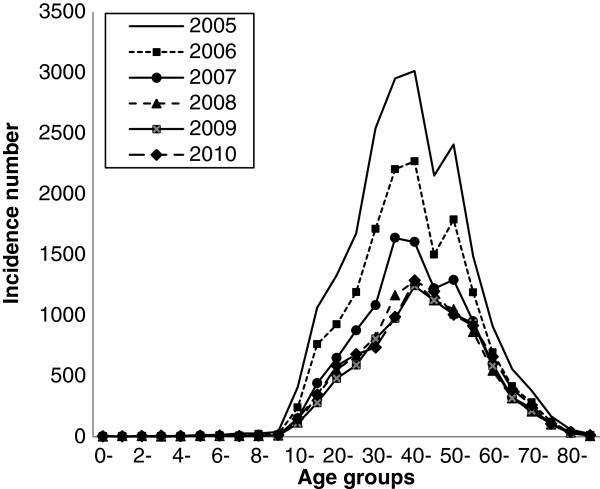
Age distribution of HFRS in China from 2005 to 2010.

The total number of HFRS cases from 2005 to 2010 in China included in our analyses was 75 434. Cases were reported in 28 provinces, autonomous regions and municipalities; no cases were reported in the Xizang autonomous regions, Qinghai or Hainan provinces during these six years. The ratio of male to female cases was approximately 3.15:1 (57 272/18 162); there nas been no change in this proportion in recent years. The majority of HFRS cases occurred in adults aged 30 to 55 (68.3%; 51 527/75 434). This pattern was similar in all six years evaluated (Figure 1).

The proportion of HFRS cases among individuals targeted by EPI (16–60 years of age) showed a decrease with each year that the immunization program was conducted (Table 1). The proportion of HFRS cases in the non-EPI-targeted (non-immunized) age group increased significantly during the EPI years.

**Table 1:** HFRS cases among the EPI-targeted age group and non-targeted age group in 2005–2010 for all provinces in China

	**Total number of cases**	**Number of cases**		
**Year**		**16–60†**	**<16**	**>60**
2005	21 281	18 501 (86.9)	911 (4.3)	1869 (8.8)
2006	15 429	13 479 (87.4)	566 (3.7)	1386 (9.0)
2007	11 248	9790 (87.0)	302 (2.7)	1156 (10.3)
2008^*^	9227	7890 (85.5)	254 (2.8)	1083 (11.7)
2009^*^	8842	7497 (84.8)	226 (2.6)	1119(12.7)
2010^*^	9407	7703 (81.9)	322 (3.4)	1382 (14.7)

***Note.*** *EPI conducted; †EPI-targeted. Data presented as% of total HFRS cases/yr.

The *χ*2 test results for HFRS annual number of cases of all provinces for the EPI-targeted and non-targeted age groups indicated that there was no significant difference in number of cases during the years between 2005 and 2007 (*χ*2 = 1.449,P = 0.485), prior to the implementation of the EPI program. In contrast, a significant difference was found when cases from any EPI year (2008–2010) were compared with those from any non-EPI year (2005–2007) (*χ*2 = 51.095, P <0.05).

The data evaluated from 2005–2010 included the two non-vaccination age groups: children and early- to mid-adolescents (<16 years), and older adults (>60 years). When the <16 age group HFRS cases were compared between non-EPI years (2005–2007) and EPI years (2008–2010), it was found that the numbers did not change significantly (P = 0.142). In contrast, the number of HFRS cases in the >60 age group was significantly different (P < 0.05) between non-EPI and EPI years. HFRS cases in the older non-vaccinated population (>60 years) rose from 8.8% in 2005 to 14.7% in 2010.

When the nationwide HFRS cases were split among the high-incidence, EPI-targeted provinces and the low-incidence, non-EPI provinces, it was found that they displayed different age distribution patterns during the study period. The proportion of HFRS reported cases for the 16–60 age groups actually rose (from 82.9% in 2009 to 87.1% in 2010) in the non-EPI regions (Table 2).

**Table 2:** HFRS cases among EPI-targeted age group and non-targeted age group from 2005–2010 in Non-EPI and EPI provinces

		**Total number of cases**	**Incidence per 100 000 population**	**Number of cases (%)**		
	**Year**			**16-60 years of age**	**<16 years of age**	**>60 years of age**
Non-EPI provinces	2005	669	0.22	614(91.8)	21(3.1)	34(5.1)
	2006	432	0.14	392(90.7)	16(3.7)	24(5.6)
	2007	230	0.08	201(87.4)	10(4.3)	19(8.3)
	2008	219	0.07	193(88.1)	8(3.7)	18(8.2)
	2009	199	0.06	165(82.9)	9(4.5)	25(12.6)
	2010	194	0.06	169(87.1)	5(2.6)	20(10.3)
Provinces targeted for EPI since 2008	2005	17 402	4.93	15 177(87.2)	742(4.3)	1483(8.5)
	2006	12 137	3.42	10 680(88.0)	436(3.6)	1021(8.4)
	2007	8374	2.34	7394(88.3)	208(2.5)	772(9.2)
	2008^*^	6959	1.93	5975(85.9)	184(2.6)	800(11.5)
	2009^*^	6378	1.76	5464(85.7)	166(2.6)	748(11.7)
	2010^*^	6995	1.92	5709(81.6)	261(3.7)	1025(14.7)
	2005	3210	0.51	2711(84.5)	148(4.6)	351(10.9)
	2006	2860	0.46	2408(84.2)	112(3.9)	340(11.9)
	2007	2644	0.42	2195(83.0)	84(3.2)	365(13.8)
	2008	2049	0.32	1723(84.1)	62(3.0)	264(12.9)
	2009^**^	2265	0.36	1867(82.5)	52(2.3)	346(15.3)
	2010^**^	2218	0.35	1825(82.3)	56(2.5)	337(15.2)

Note.^*^Statistically significant decrease (p < 0.05) in the number of cases from pre-vaccine years (2005–2007). ^**^Statistically significant decrease in the number of cases (p < 0.05) from pre-vaccine years (2005–2008).

### Analysis of high-incidence, EPI-targeted provinces

Despite the implementation of EPI, HFRS cases have remained mainly concentrated in Northeast and East China, where the seven high-incidence provinces are located. In 2010, these seven provinces accounted for 74.3% (6978/9407) of the total cases reported in China, and this situation had changed very little over the other five years: 81.7% in 2005 (17 383/21 281), 78.6% in 2006 (12 129/15 429), 74.5% in 2007, (8380/11 248), 75.4% in 2008 (6958/9227) and 72.2% in 2009 (6380/8842).

**Figure 2:**
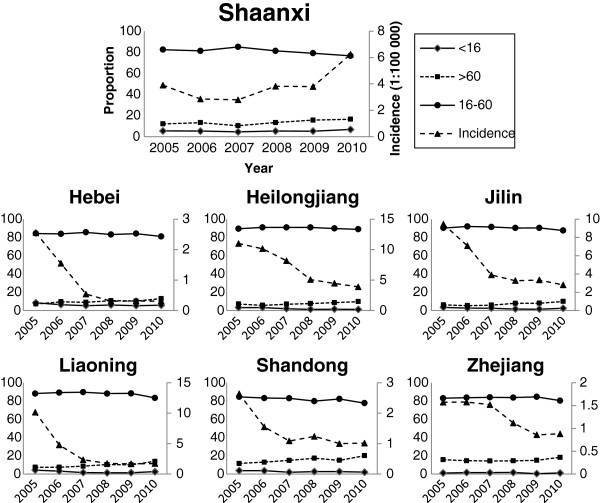
Proportion of HFRS among different age groups and the incidence per 100 000 population per province in seven EPI-target provinces from 2005 to 2010.

Over the last three years the number of HFRS cases in Shaanxi province has steadily increased. In 2010, the total annual number of reported cases (2356) had increased by 65% over the 2009 case number (1428), and had the highest number of HFRS cases in any year since 2005 [5]. This might be partly explained by well-known periodic changes in zoonoses and some social or environmental factors. Like other high-incidence provinces, the proportion of cases in the 16–60 age groups has declined since 2008 when EPI was first implemented in Shaanxi. However, the proportion of HFRS cases among the >60 age group has been substantially higher than in other high-incidence provinces. From 2009 to 2010 the HFRS cases in >60 year old Shaanxi residents increased by 67.2% (413 to 247) (Figure 2).

Heilongjiang, Jilin, Liaoning, Shandong, Hebei and Zhejiang provinces showed a steady decline in total HFRS cases since 2005, but the slope of this downward trend has decreased in recent years. Moreover, the incidence per 100 000 population in Liaoning, Shandong and Zhejiang provinces rebounded slightly in 2010. In each of these six provinces, the proportion of cases in 2010 among the respective 16–60 age groups decreased (ranging from 0.7% in Heilongjiang to a high of 8.1% in Shandong) and the proportion of cases in the >60 age groups increased (ranging from 26.7% in Zhejiang to 45.9% in Liaoning). The proportion of HFRS cases among the respective <16 age groups did not experience any obvious changes in the years examined (Figure 2).

## Discussion

As a zoonosis, HFRS shows periodic variations, which may be one of the reasons why the number of cases decreased in the years 2005–2007. HFRS has also been shown to be associated with geographical locale, employment type, living (hygiene) conditions and the epidemiology of the rodent-vector [3,24,25]. The national implementation of a comprehensive prevention and control strategy, involving rodent control, environmental management and vaccination, has corresponded to a remarkable decline in incidence of HFRS in China.

In order to reduce the incidence further, the EPI was implemented in 2008. When the proportions of cases among the different HFRS age groups were analyzed, it was found that the age distribution had changed in recent years in the EPI regions. The group that was targeted by EPI (16–60 years of age) experienced a significant decrease in the proportion of HFRS cases for each year that EPI was implemented, suggesting that vaccination might have played a considerable role in China’s HFRS prevention and control. Further analysis revealed that the proportion of cases in children less than 16 years old was relatively unaffected during this period. Moreover, this stable profile remained when only the <16 years old, non-EPI-targeted HFRS cases were analyzed (<10 years old vs. 10–16 years old, for all years examined; data not shown).

We were intrigued to discover, however, that the proportion of HFRS cases in the population over 60 years old in EPI-targeted provinces had increased annually from 2008 to 2010. In fact, Shandong province had 20.2% of its HFRS cases in the >60 age group in 2010. In stark contrast, the proportion of cases in the over 60 age group residing in non-EPI-targeted provinces had declined in 2010.

The observation that the proportion of HFRS cases over 60 years of age has risen in EPI-targeted regions may be explained by the following reasons. In recent years, more young people from rural areas have travelled to the cities for work, increasing the necessity for the elderly who have stayed behind to spend longer hours working in the fields. The elderly then represent the population most in contact with rodents and potential transmission of Hantavirus. Furthermore, the gradual extension of average life expectancy may have increased the proportion of individuals in the >60 age group who are still alive and working and potentially coming into contact with Hantaviruses.. Another potential explanation may be that China Pharmacopoeia defined the HFRS bivalent vaccine-target group as 16–60 years old, and over the dozen years since HFRS vaccination the current >60 years age group might not have had sufficient opportunity for immunization.

## Conclusions

The recent trend showing an increase in the proportion of HFRS cases in individuals over 60 years of age has occurred in regions undergoing rodent-based prevention strategies and environmental remediation and in which comprehensive public health education is widely available. Unfortunately, these non-vaccine based eradication strategies have not been sufficient to protect this older population from HFRS. Therefore, we recommend that the age limit for EPI vaccination be extended beyond 60 years old, as we believe it would be beneficial to HFRS control and prevention in China.

## Competing interests

The authors declare that they have no competing interests.

## Authors’ contributions

XH was involved in data collection, data analysis and drafting of the manuscript. SW conceived and designed the study, also participated in interpreting the data. XH assisted with reviewing and revision the manuscript. XW conducted the analysis and drafted the manuscript. All authors read and approved the final manuscript.

## Pre-publication history

The pre-publication history for this paper can be accessed here:

http://www.biomedcentral.com/1471-2458/13/394/prepub
